# Enhanced Tangential Flow Filtration of Precipitated Proteins Using Screened Membrane Cassettes

**DOI:** 10.3390/membranes15080245

**Published:** 2025-08-11

**Authors:** Zachary Badinger, Ali Behboudi, Andrew L. Zydney

**Affiliations:** Department of Chemical Engineering, The Pennsylvania State University, University Park, PA 16802, USA; zsb5096@psu.edu (Z.B.); aub793@psu.edu (A.B.)

**Keywords:** tangential flow filtration, screened cassette, monoclonal antibody, protein precipitation, bioprocessing

## Abstract

Background: Recent advances in cell culture have led to significant increases in monoclonal antibody (mAb) titers, opening a new window of opportunity for developing a fully continuous downstream purification process based on the selective precipitation of the mAb from harvested cell culture fluid, with the precipitate dewatered and washed using single-pass tangential flow filtration (SPTFF) with microfiltration membranes. Methods: Experiments were performed with precipitates of human serum immunoglobulin G formed using ZnCl_2_ and polyethylene glycol, both with and without added disodium malonate. SPTFF was conducted in both hollow fiber and screened cassette modules, with the critical flux identified using flux-stepping experiments. Results: Critical fluxes as high as 250 L/m^2^/h were obtained in the screened cassette, significantly higher than what was possible in hollow fiber modules. A two-stage system was designed that provided up to 85% conversion in a single pass. This system could be operated continuously for 24 h with 80% conversion at a filtrate flux of 144 L/m^2^/h without any significant fouling. Conclusions: The results demonstrate the potential of using screened membrane cassettes for the continuous/intensified processing of precipitated proteins like monoclonal antibodies.

## 1. Introduction

Monoclonal antibodies (mAbs) are used to treat a variety of diseases, including many cancers and autoimmune disorders [1]. The mAb market size has grown to over USD 210 billion per year and is expected to continue increase at an annual growth rate of more than 10% over the next decade [2]. One of the significant challenges limiting the availability of lifesaving mAb products in many parts of the world is cost, with many mAbs running more than USD 50,000 per patient per year. This situation will become even more critical as mAbs are developed for very high-volume applications, e.g., in the treatment of Alzheimer’s (with a current patient population of more than 50 million worldwide [3]) or hypercholesterolemia.

Notable progress has been made in enhancing mAb manufacturing processes since the commercialization of the first mAb product in 1986. Advances in upstream cell culture using Chinese hamster ovary (CHO) cells have led to titers well above 10 g/L compared to the 0.1 g/L that was common only 30 years ago [4,5]. These increases in titer have placed significant pressure on the downstream purification process, leading to a “bottleneck” associated with the limited capacity of the protein A affinity chromatography resins that are part of the current platform process for commercial mAb production [6].

Protein precipitation is an attractive alternative to bind-and-elute protein A chromatography, with high-mAb titers providing a strong thermodynamic driving force for mAb precipitation [7,8,9,10]. A variety of precipitants have been demonstrated to provide high mAb yield and good selectivity, including the combination of ZnCl_2_ as a reversible crosslinking agent and polyethyleneglycol (PEG) as a volume exclusion agent [8,11]. The resolubilized mAb shows no loss in biological activity. In addition, protein precipitation is readily performed in tubular flow reactors, which would greatly facilitate the development of a low-cost fully continuous process for mAb manufacturing [12,13].

The precipitated mAb can be effectively dewatered and washed to remove soluble host cell protein (HCP) impurities using either centrifugation or tangential flow filtration (TFF). One distinct advantage of TFF is that single-pass tangential flow filtration (SPTFF) can be easily designed to provide continuous dewatering/washing of protein precipitates [8,13]. However, with one exception, all previous studies examining TFF for protein precipitates have been performed using hollow fiber membrane modules, with the open fiber lumen assumed to be particularly attractive for processing the large (>10 µm diameter) precipitate particles. Minervini et al. [14] did compare the performance of hollow fibers and open channel cassettes, with the cassettes showing less “clogging” than the hollow fiber modules. However, the conversion in the open channel cassette, defined as the ratio of the permeate to feed flow rate, was only 6%, which is far too low to achieve any significant dewatering or washing of the precipitate in a single pass. We are not aware of any data on the use of screened membrane cassettes for processing protein precipitates.

It is well established that screened cassettes can provide enhanced mass transfer during ultrafiltration due to the “mixing” associated with the complex flow in and around the screen elements [15]. For example, Binabaji et al. [16] reported filtrate flux values in a Pellicon 3 screened cassette that were approximately 3 times larger than that in a hollow fiber module during the ultrafiltration of a 40 g/L mAb solution. A similar improvement in flux for screened cassettes was reported by Mendes et al. [17] during purification of adeno-associated virus (AAV). However, Bodik et al. [18] found very similar performance of flat sheet and hollow fiber membranes in the treatment of municipal wastewater. In contrast, Mundle et al. [19] compared the performance of hollow fiber and screened cassettes for the concentration of a herpes simplex virus 2 (HSV-2) vaccine candidate by TFF. The screened cassette showed unacceptably low yields under all conditions (<40%), while the hollow fiber modules were able to effectively concentrate the virus particles with close to 100% step yield. The authors hypothesized that the poor performance of the screened cassette was due to the adverse effects of hydrodynamic shear stresses on the viral vaccine, although no detailed analysis of the underlying mechanisms was provided. Similarly, Reid et al. [20] reported better performance of hollow fiber membranes than flat sheet cassettes for the concentration and washing of both E. coli cell suspensions and E. coli lysate. There have been no prior studies on the TFF of precipitated proteins using screened membrane cassettes.

The objective of this study was to examine for the first time the potential of using a screened TFF cassette for processing protein precipitates by SPTFF with the high conversions needed for effectively dewatering/washing the precipitate. Data were obtained using human serum immunoglobin G (hIgG), which is of interest as a therapeutic agent to treat patients with compromised immune systems and has been extensively employed as a lower cost model for mAb products. Precipitates were formed using ZnCl_2_ and PEG, both with and without disodium malonate, which has previously been shown to significantly improve the filterability of hIgG precipitates in hollow fiber modules [21].

## 2. Materials and Methods

### 2.1. Materials

Lyophilized human serum immunoglobin G (hIgG) powder was obtained from NovaBiologics (Oceanside, CA, USA) and stored at 4 °C. hIgG was used as a model protein due to its ready availability and its extensive previous use as a model for monoclonal antibody products [21,22,23]. MES (2-morpholineoethanesulphonic acid) with a pK_a_ of 6.15 was purchased from Thermo Fisher Scientific (Waltham, MA, USA). Solutions of zinc chloride (ZnCl_2_, 0.1 M), polyethylene glycol (PEG, Mw ~3350 Da), dibasic disodium malonate (DSM, ≥97.0%), and HCl were all obtained from Sigma-Aldrich (St. Louis, MO, USA).

**Figure 1:**
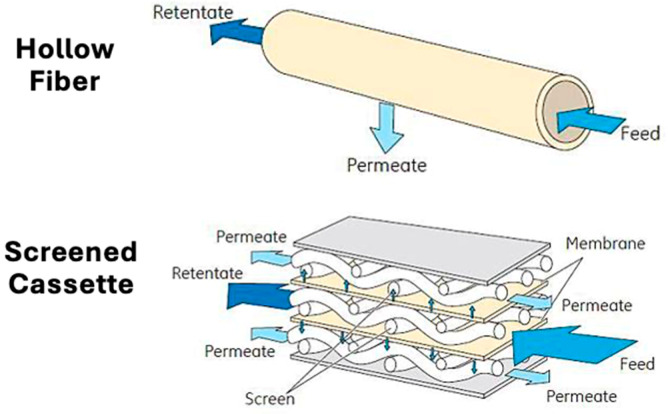
Schematic comparing the open channel hollow fiber membrane module and the screened membrane cassette. Adapted from [24].

Tangential flow filtration experiments were performed with both flat sheet cassettes and hollow fiber membrane modules, as shown schematically in Figure 1. Pellicon^®^ XL50 cassettes with C-screen and 50 cm^2^ of Durapore^®^ polyvinylidene fluoride membranes with 0.2, 0.45, or 0.65 µm pore sizes were purchased from MilliporeSigma (Burlington, MA, USA). MidiKros^®^ 0.2 µm pore size polyethersulfone hollow fiber membranes (1 mm inner diameter, 20 cm long, 88 cm^2^ effective area) were purchased from Repligen Corporation (Rancho Dominguez, CA, USA). All membranes were cleaned after each filtration experiment with 0.5 M NaOH at 35–40 °C for at least 30 min and then stored in 0.1 M NaOH. Water permeability measurements were obtained before each filtration experiment to verify that the membrane was undamaged and that there was no irreversible fouling (permeability within ±10% of that for the clean module).

### 2.2. hIgG Precipitation

Stock solutions of hIgG were prepared using 50 mM MES buffer at pH 6.4. Recent studies have shown that disodium malonate can significantly increase the packing density of the precipitate particles and, in turn, enhance their filterability during single-pass tangential flow filtration [21]. Thus, experiments were also performed with 20 mM disodium malonate added. The resulting protein solutions (before precipitation) were filtered through 0.2 µm pore size polyethersulfone flat sheet membranes (MilliporeSigma, Burlington, MA, USA) to remove any undissolved protein.

hIgG precipitation was performed in a series of tubular flow reactors with static mixers (Koflo Corporation, Cary, IL, USA) used to maintain a relatively uniform residence time distribution. Precipitation was performed using ZnCl_2_ as a nucleating agent and PEG as a volume exclusion agent based on recent studies [11,13]. The hIgG solution was fed to the first reactor at a flow rate of 5 mL/min and mixed with a 1 mL/min stream of 0.1 M ZnCl_2_ via a Y-connector at the inlet to the tubular reactor. A 17.5 w/v% PEG solution was added at a flow rate of 4 mL/min through a second Y-connector to the inlet of the second reactor, resulting in a final solution containing 5 g/L hIgG, 10 mM ZnCl_2_, and 7 w/v% PEG. Flow rates were controlled with Master flex peristaltic pumps (Cole-Parmer Instrument, Vernon Hills, IL, USA). The precipitated protein was collected and stored for 12 h at 4 °C to prevent bacterial growth.

The size distribution for the precipitated hIgG was evaluated by laser diffraction using a Mastersizer 3000 (Malvern Panalytical, Worcestershire, United Kingdom) with a Hydro MV wet dispersion attachment. The sample tank was initially filled with a solution of 10 mM ZnCl_2_ and 7 w/v% PEG to prevent redissolution of the hIgG precipitate during analysis. The size distributions were determined by the Mie scattering theory directly by Mastersizer 3000 software (version V3.62).

### 2.3. Critical Flux

The critical flux, also known as the sustainable flux, was evaluated using the flux-stepping procedure described in the literature [8,14]. The modules were initially flushed with MES buffer, with or without disodium malonate, to match the composition of the hIgG precipitate. The precipitate solution was pumped from a 250 mL reservoir through the module using a Masterflex peristaltic pump, with a second pump placed at the retentate outlet to control the degree of filtration. The system was operated in total recycle mode, with both the retentate and permeate streams recycled to the feed reservoir to minimize the required feed volume. The feed and retentate pressures were monitored using digital pressure gauges (Ashcroft, Stratford, CT, USA), while the permeate exit was left open to atmospheric pressure. The transmembrane pressure (TMP) was calculated as

(1) $$TMP=\frac{P_{F}+P_{R}}{2}-P_{p}$$

where P_F_, P_R_, and P_p_ are the pressures of the feed, retentate, and permeate, respectively. The retentate flow rate was decreased stepwise every 25 min, using steps of 1 mL/min (corresponding to an increase in the permeate flux of 12 L/m^2^/h in the cassette and 7 L/m^2^/h in the hollow fiber). The critical flux was evaluated as the maximum permeate flux at which the TMP remained stable over a 25 min interval, with unstable operation defined as the conditions that first gave a TMP gradient of at least 0.1 kPa/min.

Multi-stage filtration experiments were run using two Pellicon^®^ XL50 cassettes in series, with the outlet retentate flow from the first module used as the feed to the second module. The overall conversion was controlled using a pump on the retentate exit, with the second (downstream) permeate ports of both cassettes kept open to the atmosphere.

Long-time filtration experiments were performed in total recycle mode at fixed feed and retentate flow rates based on results from the flux stepping experiments. Data were obtained over 24 h of continuous operation using a 1 L feed reservoir.

## 3. Results

### 3.1. Module Geometry

**Figure 2:**
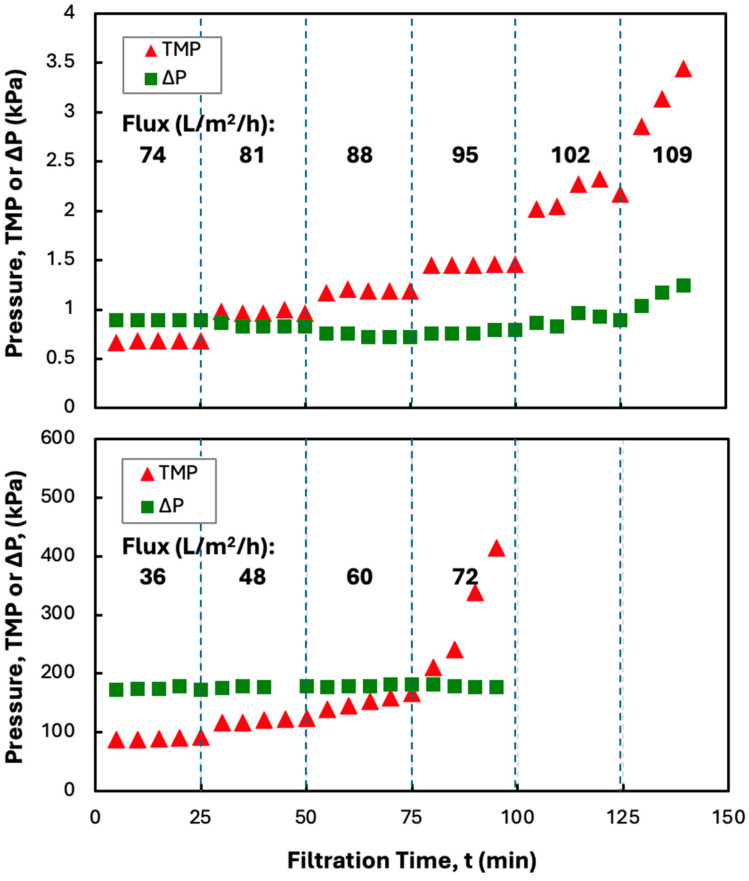
Flux-stepping experiments with MidiKros^®^ hollow fiber (**top panel**) and Pellicon^®^ XL50 screened cassette with a 0.2 µm Durapore membrane (**bottom panel**) for 5 g/L hIgG precipitates generated in 50 mM MES buffer at pH 6.4 with 10 mM ZnCl_2_ and 7 w/v% PEG (no disodium malonate). Filtration was performed at a feed flow rate 30 mL/min. Flux values are shown in bold for each time interval.

Previous studies have shown that protein precipitates can be effectively dewatered and washed using SPTFF employing microfiltration membranes [8,12,13]. Continuous processing can be achieved by operating the filtration module below the critical flux, i.e., under conditions where fouling is insignificant. A series of flux-stepping experiments were thus performed to evaluate the critical flux for precipitates formed using 5 g/L solutions of hIgG in 50 mM MES buffer, using ZnCl_2_ and PEG as the precipitating agents. Data for the TMP and the feed-side pressure drop (∆P = P_F_ *−* P_R_) are shown in Figure 2 for experiments performed at a feed flow rate of 30 mL/min in both the Pellicon*^®^* XL50 screened cassette (using the 0.2 µm pore size Durapore membrane) and the MidiKros*^®^* hollow fiber module. The TMP in the hollow fiber modules remained completely stable over each 25 min interval for filtrate flux up to 95 L/m^2^/h, with the TMP gradient being below 0.1 kPa/min even at a filtrate flux of 102 L/m^2^/h. The feed-side pressure drop was stable throughout the experiment, with values below 1.5 kPa, suggesting that there was minimal fiber blockage even at high filtrate flux. All the flux-stepping experiments performed were repeated at least twice, with excellent agreement between the results; this is discussed further subsequently. The error bars on the pressure measurements were all smaller than the size of the symbols and are not shown.

The TMP values in the screened cassette were 100x higher than those in the hollow fiber module, even though the clean membrane permeability differed by less than 20% (69 ± 7 L/m^2^/h/kPa for the cassette compared to 82 ± 5 L/m^2^/h/kPa for the hollow fiber module). The much higher TMP in the cassette is a direct result of the large feed-side pressure drop associated with the additional parasitic pressure losses caused by the flow in and around the screen. The higher TMP values in the screened cassette were still small enough that they would not create any issues in terms of the required pumping costs, and there was no evidence that these higher pressures caused any additional fouling, as discussed subsequently. The TMP in the screened cassette showed a small increase at a filtrate flux of 48 L/m^2^/h, with the TMP gradient exceeding 0.1 kPa/min at a filtrate flux of 60 L/m^2^/h, which is barely more than one-half the critical flux in the hollow fiber module. This was true even though the hollow fiber module operated at a slightly lower feed flux; both devices were operated at a feed flow rate of 30 mL/min, but the hollow fiber module has a slightly larger membrane area (88 cm^2^ compared to 50 cm^2^ for the Pellicon^®^ XL50 cassette). Instead, the lower critical flux in the screened cassette is likely due to deposition/capture of the amorphous precipitate within the narrow spaces between the screen and membrane, which is completely absent in the hollow fiber module. This is likely similar to the behavior observed by Mundle et al. [19] in their analysis of TFF for the concentration of an HSV-2 vaccine product.

### 3.2. Effect of Disodium Malonate

**Figure 3:**
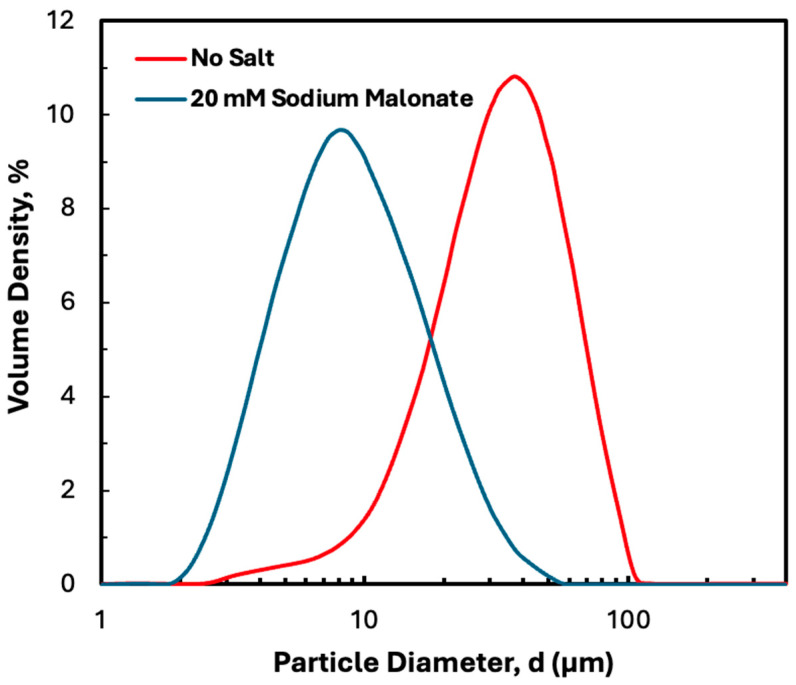
Particle size distribution determined by laser diffraction for 5 g/L hIgG precipitates formed in 50 mM MES buffer at pH 6.4 with 10 mM ZnCl_2_ and 7 w/v% PEG both with and without 20 mM disodium malonate.

As discussed in a previous publication, disodium malonate has a significant effect on the structure and properties of precipitated hIgG, although it does not affect the properties of the protein itself, as determined by circular dichroism [21]. Figure 3 shows the particle size distribution determined by laser diffraction both in the absence and presence of disodium malonate. The addition of 20 mM disodium malonate reduced the mean particle size from 49 to 11 µm, with almost no particles observed with diameter greater than 60 µm in the presence of disodium malonate. The measured size distribution was largely unaffected by SPTFF, with no evidence of any particle aggregation or dissociation due to the shear flow in either the hollow fiber or screened cassette devices. Note that disodium malonate also increases both the protein stability, based on differential scanning calorimetry and isothermal titration calorimetry measurements [21], and the packing density of the protein precipitate, based on measurements of the mass of pellet obtained after centrifugation. Previous studies have shown that the critical flux in hollow fiber modules is very well-correlated with the packing density, with higher packing densities providing significantly better filterability over a range of conditions [21,25].

**Figure 4:**
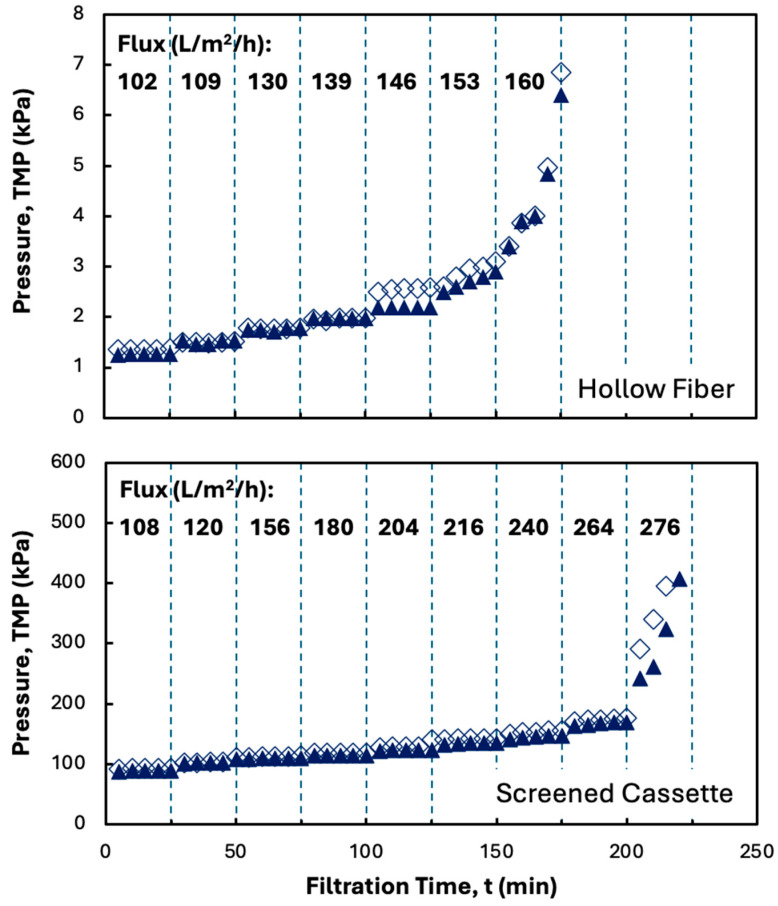
Flux-stepping experiments with the MidiKros^®^ hollow fiber (top panel) and Pellicon^®^ XL50 screened cassette with 0.2 µm Durapore membrane (bottom panel) performed using precipitated hIgG (5 g/L) generated in 50 mM MES buffer at pH 6.4 with 10 mM ZnCl_2_ and 7 w/v% PEG in the presence of 20 mM disodium malonate. Filtration was performed at a feed flow rate of 30 mL/min. Filled and open symbols in each panel are from repeat experiments under identical conditions. Flux values are shown in bold for each time interval.

The effect of disodium malonate on the filtration performance in the MidiKros^®^ hollow fiber module and Pellicon^®^ XL50 cassette is examined in Figure 4, with data shown for two repeat experiments in both the hollow fiber module and screened cassette. The results from the repeat experiments are in excellent agreement; similar results were obtained at the other conditions examined in this study. The addition of disodium malonate significantly reduced membrane fouling in both devices, with the TMP in the hollow fiber remaining stable up to a flux of 146 L/m^2^/h (nearly 50% higher than in Figure 2), while that in the cassette remained stable up to a flux of 264 L/m^2^/h, with the latter being a factor of four larger than that obtained in the absence of disodium malonate. The large increase in critical flux is due to the denser structure of the hIgG precipitate formed in the presence of disodium malonate, which likely reduces the deposition of precipitate particles on the membrane surface. This makes the additional mixing provided by the internal screen much more effective, with the critical flux in the presence of disodium malonate being 80% larger in the Pellicon^®^ XL50 than that in the hollow fiber module (the exact opposite of the behavior seen in Figure 2). It is important to note that the hollow fiber has a slightly larger membrane area than the screened cassette (88 cm^2^ to 50 cm^2^). The smaller area within the cassette does increase the loading on the membrane; however, the critical flux is still higher with the screened cassette due to the significant impact of the internal screen.

Table 1 summarizes the critical flux and conversion results obtained in the Pellicon^®^ XL50 screened cassette and the hollow fiber membrane module, both in the absence and presence of disodium malonate. In both cases, the conversion (x) is defined as

(2) $$x=\frac{q_{p}}{q_{F}}$$

where q_p_ and q_F_ are the volumetric permeate and feed flow rates, respectively. Note that a conversion of 90% corresponds to a single-pass concentration factor of 10-fold The error bars represent the range determined from repeat measurements. In the absence of disodium malonate, the hollow fiber module significantly outperforms the Pellicon^®^ XL50 cassette, having both a higher critical flux and 2.5 times larger conversion. In contrast, the Pellicon^®^ XL50 cassette and hollow fiber modules have nearly identical conversions in the presence of disodium malonate, reflecting the very different behavior observed for precipitates formed with/without added disodium malonate. In addition, the critical flux for the Pellicon^®^ XL50 cassette in the presence of disodium malonate is 70% larger than that in the hollow fiber module, meaning that one can use a much smaller screened cassette (by membrane area) to process the precipitated protein.

### 3.3. Effect of Feed Flow Rate

**Figure 5:**
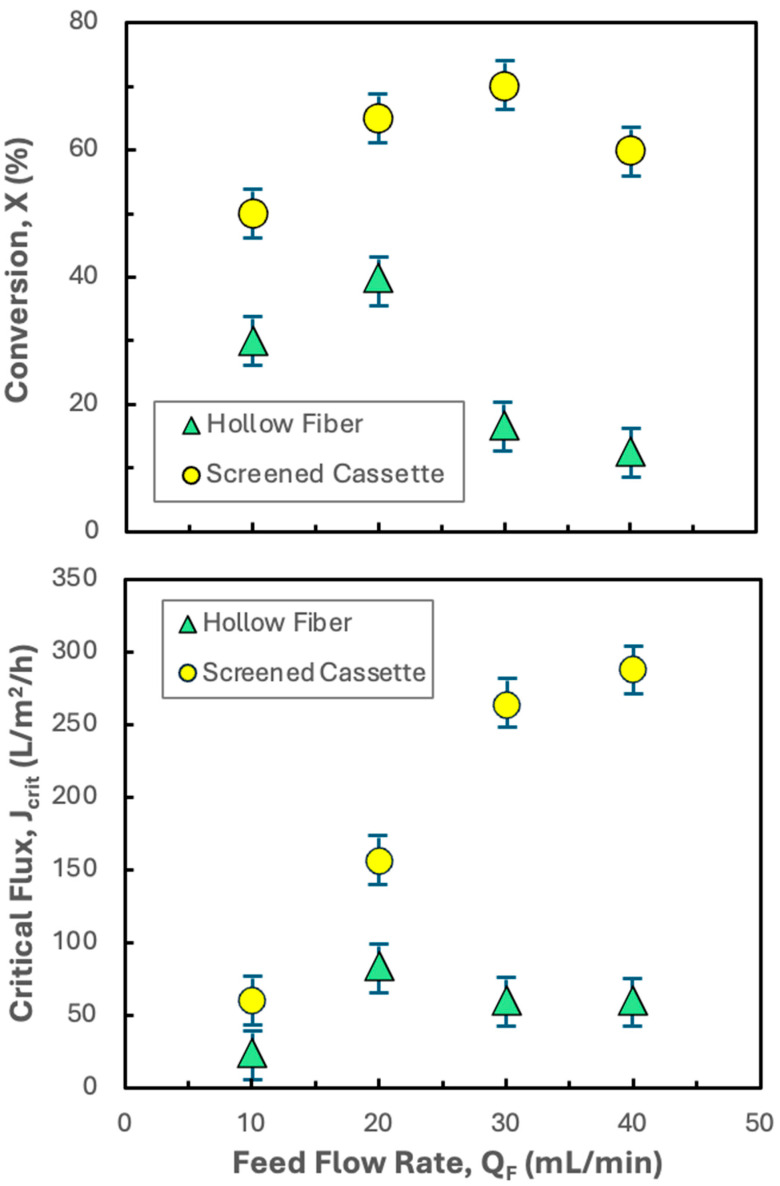
Effect of feed flow rate on the critical flux (bottom panel) and conversion (top panel) for filtration of 5 g/L hIgG precipitates generated in 50 mM MES buffer at pH 6.4 with 10 mM ZnCl_2_ and 7 w/v% PEG both alone and in the presence of 20 mM disodium malonate (DSM) using the Pellicon^®^ XL50 cassette with 0.2 µm pore size Durapore membranes. Error bars represent the standard deviation determined from repeat experiments performed in the hollow fiber and screened cassettes at each feed flow rate.

Although the critical flux and conversion results for the Pellicon^®^ XL50 at a feed flow rate of 30 mL/min are attractive, it would be desirable to operate at even higher conversions to achieve a greater single-pass concentration factor. Figure 5 shows the effects of the feed flow rate on both the critical flux and conversion in the Pellicon^®^ XL50 for hIgG precipitates formed in the presence and absence of 20 mM disodium malonate. In the absence of disodium malonate, the critical flux shows a maximum of only 84 L/m^2^/h at a feed flow rate of 20 mL/min. The reduction in critical flux at higher feed flow rates is surprising given that the tangential flow should be more effective at sweeping the membrane surface as the feed flow rate increases. In contrast, the critical flux in the presence of disodium malonate increases monotonically with increasing feed flow rate, varying by more than a factor of five as the feed flow rate increases from 10 to 40 mL/min. However, the conversion goes through a weak maximum at a feed flow rate of 30 mL/min, decreasing from 70% to 60% as the feed flow rate increases from 30 to 40 mL/min (despite the increase in the critical flux). This behavior is due to the less than linear dependence of the critical flux on the feed flow rate at higher feed flow rates. Data were not obtained at either higher or lower feed flow rates, since these led to lower conversions; effective dewatering and washing of the precipitate requires high single-pass conversions to remove impurities in the permeate.

**Figure 6:**
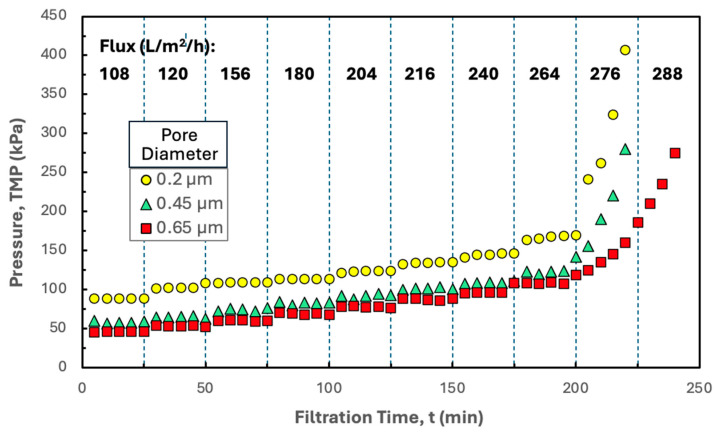
Flux-stepping experiments performed with the Pellicon^®^ XL50 cassette with 0.2, 0.45, and 0.65 µm pore size Durapore membrane performed using precipitated hIgG (5 g/L) generated in 50 mM MES buffer at pH 6.4 with 10 mM ZnCl_2_ and 7 w/v% PEG in the presence of 20 mM disodium malonate. Filtration was performed at a feed flow rate of 30 mL/min. Flux values are shown in bold for each time interval.

Additional insights into the filtration behavior were obtained by performing flux-stepping experiments with 0.20, 0.45, and 0.65 µm Durapore membranes in the Pellicon^®^ XL50 cassette using hIgG precipitates formed in the presence of disodium malonate, with the resulting TMP profiles shown in Figure 6. The critical flux was nearly independent of the pore size, suggesting that the fouling occurs primarily by deposition of the hIgG precipitate on the surface of the membrane (or in the gaps between the spacer and the membrane) and not within the depth of the porous structure. The TMP increased with decreasing pore size at all values of the filtrate flux, which is consistent with the lower permeability for the smaller pore size membranes.

### 3.4. Multi-Stage Filtration

**Figure 7:**
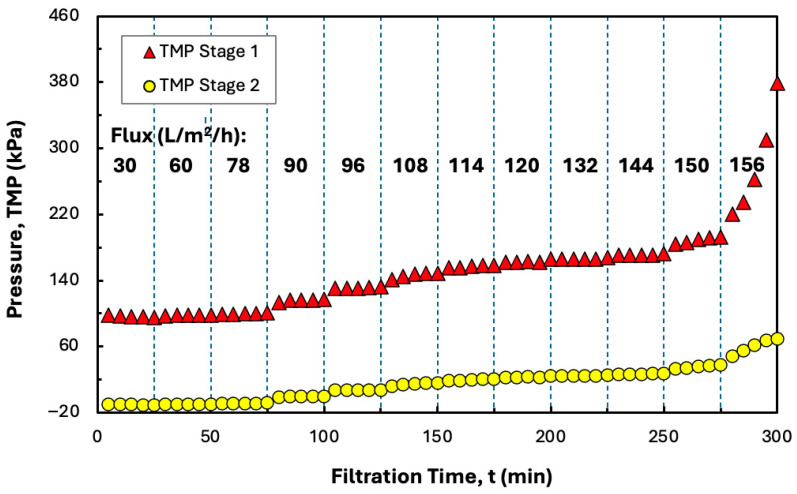
Flux-stepping experiments performed with two Pellicon^®^ XL50 cassettes in series using precipitated hIgG (5 g/L) generated in 50 mM MES buffer at pH 6.4 with 10 mM ZnCl_2_ and 7 w/v% PEG in the presence of 20 mM disodium malonate. Filtration was performed at a feed flow rate of 30 mL/min. Flux values are shown in bold for each time interval.

Since the data in Figure 5 indicates that it is not possible to increase the conversion beyond 70% in a single Pellicon^®^ XL50 cassette, flux-stepping experiments were also performed with two Pellicon^®^ XL50 cassettes in series, with the retentate from the first cassette used directly as the feed to the second cassette (without any additional pumping). The permeate flow rate was again controlled using a pump on the retentate exit. As shown in Figure 7, the TMP in the second stage was close to zero until the filtrate flux increased to 90 L/m^2^/h, with all of the permeate collected through the first stage under these conditions. Further increases in the total permeate flux resulted in flow through both stages, although the TMP in the second stage remained well below that in the first stage. The TMP became unstable at a total permeate flux of 156 L/m^2^/h, with more rapid fouling observed in the first stage.

The addition of a second stage reduced the critical flux from 264 to 150 L/m^2^/h due to the relatively small increase in the total permeate flow rate compared to the doubling of the membrane surface area. However, the two-stage configuration did provide a significantly higher conversion of 85% compared to the maximum conversion of only 70% in the single cassette. Note that this increase in conversion corresponds to a doubling of the concentration factor (from 3.3 to 6.7), which has significant implications for the dewatering and washing of protein precipitates using TFF [8,13].

### 3.5. Long-Term Filtration

**Figure 8:**
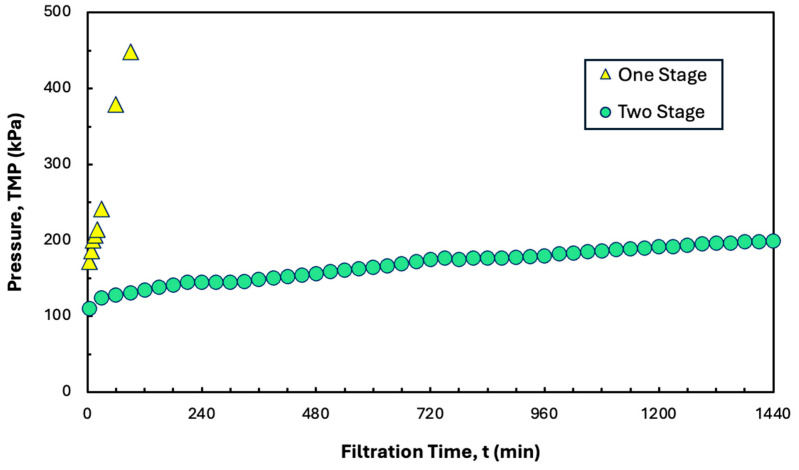
TMP profiles for long-term constant flux experiments using precipitated hIgG (5 g/L) generated in 50 mM MES at pH 6.4 with 10 mM ZnCl_2_ and 7 w/v% PEG with 20 mM disodium malonate. Filtration was performed at a feed flow rate of 30 mL/min using one- and two-stage configurations of the Pellicon^®^ XL50 cassette at conversions of 80%.

In principle, it should be possible to operate the SPTFF system continuously for an extended period of time as long as the filtrate flux is maintained below the critical flux. This was examined experimentally by performing a long-time (24 h) filtration experiment at a constant conversion of 80% using the two-stage system from Figure 7. Data were obtained with 1 L of a precipitate formed using 5 g/L hIgG in the presence of 20 mM disodium malonate using a total recycle configuration to minimize the required volume of feed. As shown in Figure 8, the overall TMP remained below 200 kPa over the 24 h experiment, with an average TMP gradient of only 3.75 kPa/h. This system could potentially be operated for a total of 5.6 days assuming linear extrapolation of the data in Figure 8 and a maximum safe operating TMP of 600 kPa. Also shown for comparison are data obtained with a single Pellicon^®^ XL50 cassette operated under identical conditions. In this case, the TMP increased rapidly to more than 600 kPa after only 120 min of operation, consistent with the system being operated at a flux of 288 L/m^2^/h, which is above the critical flux of 264 L/m^2^/h under these conditions. Note that previous studies showed that the maximum stable conversion in a hollow fiber module was only 70% [21].

## 4. Discussion

The results obtained in this study provide the first demonstration that it is possible to use screened membrane cassettes to process precipitated proteins with significantly higher filtrate flux than possible with hollow fiber devices. However, this improvement in performance is only possible when the hIgG is precipitated in the presence of disodium malonate; data obtained in the absence of disodium malonate show higher critical flux and much higher conversion in the hollow fiber module. Precipitation in the presence of disodium malonate generates smaller and more compact particles, which are much easier to filter, particularly in the complex flow geometry in the screened cassette.

The maximum conversion that could be obtained in the screened cassette was only 70%, which corresponds to a concentration factor of 3.3. However, conversions as high as 85% could be achieved using a two-stage system in which the retentate outlet from the first stage is fed directly into the second cassette. This two-stage system was able to provide 80% conversion (5-fold single pass concentration) over 24 h of continuous operation with a minimal increase in transmembrane pressure. This increase in conversion will have a dramatic impact on the extent of HCP removal for a process using a combined dewatering and countercurrent washing [13]:

(3) $$R=\left(\frac{1}{1-x}\right)\left(\frac{\alpha^{N+1}-1}{\alpha -1}\right)$$

where R is the degree of HCP removal (concentration in the feed divided by concentration after dewatering/washing the precipitate), x is the conversion in the membrane module, and α = x/(1 − x). Thus, a system with N = 3 stages and 70% conversion will provide a 71-fold reduction in HCP concentration, but this increases to R = 425 with 80% conversion and R = 8200 with 90% conversion.

The critical flux for the screened cassette was more than 250 L/m^2^/h, which is significantly higher than that reported previously for an optimized hollow fiber module with the same feed material [21]. Additional studies will be required to fully characterize the filtration mechanisms in the screened cassette to develop more effective precipitation-filtration processes for the purification of monoclonal antibodies.

## Figures and Tables

**Table 1:** Critical flux and conversion for precipitated hIgG in the presence and absence of disodium malonate in the Pellicon^®^ XL50 and hollow fiber modules at a feed flow rate of 30 mL/min.

Pellicon XL50	Critical Flux (L/m^2^/h)	Conversion	Hollow Fiber	Critical Flux (L/m^2^/h)	Conversion
No malonate	60 ± 12	0.17 ± 0.03	No malonate	100 ± 7	0.46 ± 0.03
With malonate	260 ± 12	0.70 ± 0.03	With malonate	150 ± 7	0.70 ±.0.03

## Data Availability

Data will be made available upon request.

## Abbreviations

The following abbreviations are used in this manuscript:

DSM	Disodium malonate
hIgG	Human serum immunoglobulin G
MES	2-morpholineoethanesulphonic acid
PEG	Polyethylene glycol
SPTFF	Single-pass tangential flow filtration
TFF	Tangential flow filtration
TMP	Transmembrane pressure difference
